# Phase II study of S-1, a novel oral fluoropyrimidine derivative, in patients with metastatic colorectal carcinoma

**DOI:** 10.1054/bjoc.2000.1236

**Published:** 2000-06-15

**Authors:** A Ohtsu, H Baba, Y Sakata, Y Mitachi, N Horikoshi, K Sugimachi, T Taguchi, for the S-1 Cooperative Colorectal Carcinoma Study Group

**Affiliations:** Department of Gastrointestinal Oncology/Gastroenterology, National Cancer Center Hospital East, Kashiwa; Department of Gastroenterologic Surgery, National Kyushu Cancer Center, Fukuoka; Department of Gastroenterology, Aomori Prefectural Central Hospital, Aomori; Department of Gastroenterology and Medical Oncology, Sendai Kosei Hospital, Miyagi; Chemotherapy Cancer Center, Cancer Institute Hospital, Japanese Foundation for Cancer Research, Tokyo; Department of Surgery II, Faculty of Medicine, Kyushu University, Fukuoka; Japan Society for Cancer Chemotherapy, Osaka, Japan

**Keywords:** metastatic colorectal carcinoma, tegafur, CDHP, S-1

## Abstract

This study set out to evaluate, in patients with metastatic colorectal carcinoma, the efficacy and toxicity of S-1, which contains tegafur, 5-chloro-2,4-dihydroxypyridine (CDHP) and potassium oxonate, based on a biochemical modulation of 5-fluorouracil (5-FU) targeted at inhibition of dihydropyrimidine dehydrogenase (DPD). Sixty-three patients with measurable metastatic colorectal carcinoma were enrolled into the study. None of the patients had received prior chemotherapy except for adjuvant setting. S-1 was administered orally twice daily at a standard dose of 80 mg m^–2^day^–1^for 28 days followed by a 14-day rest. This agent is continued until disease progression, unaccepted toxicity, or patient refusal. Twenty-two (35%) of the 62 eligible patients achieved PR with a 95% confidence interval of 25–48%. Five of the 10 patients with a history of adjuvant chemotherapy achieved partial remission. The median survival time was 12 months. Major adverse reactions included myelosuppressive and gastrointestinal toxicities, though their incidence of grade 3 or 4 being 13% in neutropenia and less than 10% in the others. None of the 53 patients treated as outpatients required hospitalization due to adverse reactions: These results suggest that S-1 achieves similar responses to those of infusional 5-FU plus leucovorin and shows the potential of another biochemical modulation with easily manageable toxicity. © 2000 Cancer Research Campaign


British Journal of Cancer (2000) 83(2), 141–145
© 2000 Cancer Research Campaign
doi: 10.1054/ bjoc.2000.1236, available online at http://www.idealibrary.com on IDEAL®

### Phase II study of S-1, a novel oral fluoropyrimidine derivative, in patients with metastatic colorectal carcinoma

**A Ohtsu[1], H Baba[2], Y Sakata[3], Y Mitachi[4], N Horikoshi[5], K Sugimachi[6] and T Taguchi[7], for the S-1 Cooperative Colorectal Carcinoma Study Group**

[1]Department of Gastrointestinal Oncology/Gastroenterology, National Cancer Center Hospital East, Kashiwa; [2]Department of Gastroenterologic Surgery, National Kyushu Cancer Center, Fukuoka; [3]Department of Gastroenterology, Aomori Prefectural Central Hospital, Aomori; [4]Department of Gastroenterology and Medical Oncology, Sendai Kosei Hospital, Miyagi; [5]Chemotherapy Cancer Center, Cancer Institute Hospital, Japanese Foundation for Cancer Research, Tokyo; [6]Department of Surgery II, Faculty of Medicine, Kyushu University, Fukuoka; [7]Japan Society for Cancer Chemotherapy, Osaka, Japan

**Summary** This study set out to evaluate, in patients with metastatic colorectal carcinoma, the efficacy and toxicity of S-1, which contains tegafur, 5-chloro-2,4-dihydroxypyridine (CDHP) and potassium oxonate, based on a biochemical modulation of 5-fluorouracil (5-FU) targeted at inhibition of dihydropyrimidine dehydrogenase (DPD). Sixty-three patients with measurable metastatic colorectal carcinoma were enrolled into the study. None of the patients had received prior chemotherapy except for adjuvant setting. S-1 was administered orally twice daily at a standard dose of 80 mg $m^{-2}$ $day^{-1}$ for 28 days followed by a 14-day rest. This agent is continued until disease progression, unaccepted toxicity, or patient refusal. Twenty-two (35%) of the 62 eligible patients achieved PR with a 95% confidence interval of 25–48%. Five of the 10 patients with a history of adjuvant chemotherapy achieved partial remission. The median survival time was 12 months. Major adverse reactions included myelosuppressive and gastrointestinal toxicities, though their incidence of grade 3 or 4 being 13% in neutropenia and less than 10% in the others. None of the 53 patients treated as outpatients required hospitalization due to adverse reactions: These results suggest that S-1 achieves similar responses to those of infusional 5-FU plus leucovorin and shows the potential of another biochemical modulation with easily manageable toxicity. © 2000 Cancer Research Campaign

**Keywords:** metastatic colorectal carcinoma; tegafur; CDHP; S-1

5-FU remains as the mainstay treatment for metastatic colorectal carcinoma. A combination of 5-FU with leucovorin has received widespread acceptance in the treatment regimens for this disease, with a superior response rate than that of 5-FU alone (Advanced Colorectal Cancer Meta-Analysis Project, 1992). However, even in this regimen chemotherapy has only palliative impact for metastatic colorectal carcinoma. Issues regarding cost-effectiveness have been recently addressed in the field of medical oncology and will be unavoidable in the near future (DeMario et al, 1998). Under these circumstances, oral chemotherapy has become a promising alternative in converting inpatients to outpatients and in reducing times to visit a hospital. Although the economical benefit depends on the market prices of oral agents, these agents can provide a chance to reduce the medical costs. During the period from the 1970s to the 1980s an oral fluorinated pyrimidine, a combination of uracil and tegafur (UFT), was originally developed in Japan and evaluated in Japanese clinical trials (Takiuchi et al, 1998). Uracil is observed to inhibit the activity of hepatic DPD, a key enzyme in 5-FU catabolism, thus leading to increased 5-FU levels when tegafur is administered together with uracil (Ikenaka et al, 1979). There followed widespread use of the agent by Asian physicians, especially for gastrointestinal malignancies (Takiuchi et al, 1998). However, because methodology and quality assurance of the clinical trial were immature at that time, the true impact of the agent was not assessed and is still uncertain. UFT was re-evaluated outside Japan as a single agent as well as in combination with leucovorin, with promising results (Malik et al, 1990; Pazdur et al, 1994).

*Received 16 July 1999*
*Revised 25 November 1999*
*Accepted 10 March 2000*

*Correspondence to*: A Ohtsu

S-1 is a new oral fluorinated pyrimidine developed by Taiho Pharmaceutical Co Ltd (Tokyo, Japan). The agent contains tegafur, CDHP and potassium oxonate in a molar ratio of 1:0.4:1, based on a biochemical modulation of 5-FU (Shirasaka et al, 1996). CDHP exhibits a 180-fold higher activity in inhibiting DPD than that of uracil in vitro (Tatsumi et al, 1987). Potassium oxonate inhibits phosphorylation of 5-FU by orotate pyrimidine phosphoribosyl transferase in the digestive tract (Houghton et al, 1979). The levels of 5-fluorouridine 5′-monophosphate and 5-FU incorporated into RNA are reduced to approximately 30% only in the small intestine, while the decrease is limited to 0–20% in bone marrow and tumour tissue (Shirasaka et al, 1993). Another experiment in rats bearing subcutaneous Yoshida Sarcoma cells showed that S-1 tended to prolong the concentration of 5-FU in plasma and tumour tissue more than an equitoxic dose of UFT, with less gastrointestinal toxicity (Takechi et al, 1997).

Based on the promising preclinical results, a phase I study of the agent was conducted in Japan. The study concluded that the maximum allowable dose of the agent was 75 mg $body^{-1}$ twice-daily for 28 consecutive days followed by a 14-day rest period, with dose-limiting toxicity of leucopenia (Taguchi et al, 1997).

Excellent activity against gastric cancer was achieved in the subsequent early and late phase II study, which resulted in response rates of approximately 50% in both studies, with minimal toxicity (Sugimachi et al, 1999; Sakata et al, 1998). For colorectal carcinoma, the response was only modest with a rate of 17% in early phase II study. However, the response rate was 25% in patients without prior chemotherapy, warranting further research in relation to this disease. Since the rate of discontinuation due to adverse reactions was markedly reduced for patients given 90 mg $m^{-2}$ $day^{-1}$ or less, 80 mg $m^{-2}$ $day^{-1}$ was recommended as the standard dose. The results of the following late phase II study are described in this paper.

### PATIENTS AND METHODS

### Patient eligibility

Patients eligible for this study were required to show histologically proven colorectal carcinoma with measurable or evaluable lesions. No prior chemotherapy or radiotherapy except for adjuvant chemotherapy completed at least 6 months before selection was allowed. Patients were required to have 2 or better performance status in Eastern Cooperative Oncology Group scale with a life-expectancy of 3 months or longer and to be younger than 75 years. Eligibility also required adequate organ functions: haemoglobin $\geq$ 9.0 g $dl^{-1}$; WBC $\geq$ 4000–12 000 $\mu l^{-1}$; platelets $\geq$ 100 000 $\mu l^{-1}$; AST and ALT $\leq$ 100 IU $l^{-1}$; serum alkaline phosphate within twice the normal upper limit; serum bilirubin $\leq$ 1.5 mg $dl^{-1}$; creatinine within normal upper limit; and written informed consent from the patients. Only patients that were fit enough to receive chemotherapy, with no other cancers, were eligible for this study. This study was approved by each institutional review board and was conducted in accordance with good clinical practice guideline in Japan.

### Treatment schedule

The patients were assigned on the basis of body surface area to receive one of the following doses twice daily, after breakfast and dinner: body surface area < 1.25 $m^2$, 40 mg; < 1.50 $m^2$, 50 mg; $\geq$ 1.50 $m^2$, 60 mg. S-1 was administered at the respective dose for 28 days, followed by a 2-week rest period. This schedule was repeated every 6 weeks until the occurrence of disease progression, unacceptable toxicities, or patient's refusal. The dose was reduced by 20 mg $day^{-1}$ if grade 3 or higher haematological or grade 2 or higher non-haematological toxicity was seen in the previous course. Patients who required more than 4 weeks of rest to recover from any toxicity other than alopecia or skin toxicity were retired from the treatment. No prophylactic use of anti-emetic agents was allowed. Compliance was assessed by patient interviews with each investigator, using a schedule calendar with regular monitoring.

### Evaluation of response and toxicity

Antitumour activity was evaluated in accordance with the general rule edited by the Japanese Research Society for Colorectal Carcinoma based on WHO criteria (Japanese Research Society for Cancer of Colon and Rectum, 1994). Briefly, a complete response (CR) was defined as the complete disappearance of all measurable and assessable diseases for a minimum of 4 weeks. A partial response (PR) was defined as a 50% or more reduction in the sum of the products of the longest diameter of measurable disease for a minimum of 4 weeks. Stable disease (SD) was defined as the failure to observe a partial or complete response and progressive disease for at least 4 weeks. Progressive disease (PD) was defined as a 25% or more increase in the sum of the products of the longest diameter of measurable disease or the appearance of new lesions. Objective responses were confirmed by an external review committee consisting of five oncologists.

Toxicity was evaluated according to the toxicity criteria of the Japan Society for Cancer Therapy, based on modifications of the WHO criteria (Japan Society for Cancer Therapy, 1993).

**Table 1** Patient characteristics

| | | No. of patients |
|---|---|---|
| Total eligible patients | 62 | |
| Primary site | | |
| | Colon | 43 |
| | Rectum | 19 |
| Sex | | |
| | Male | 37 |
| | Female | 25 |
| Age (years) | | |
| | Median | 62 |
| | Range | 27–74 |
| ECOG performance status scale | | |
| | 0 | 36 |
| | 1 | 19 |
| | 2 | 7 |
| Initial dosage (mg $day^{-1}$) | | |
| | 80 | 4 |
| | 100 | 25 |
| | 120 | 33 |
| Prior surgical resection (primary) | | |
| | Yes | 48 |
| | No | 14 |
| Adjuvant chemotherapy | | |
| | Yes | 10 |
| | No | 52 |

ECOG = Eastern Cooperative Oncology Group

### Statistics

The sample size for the study was calculated from an expected response rate of 20% with an $\alpha$ and $\beta$ error of 0.05 and 0.2, respectively. Therefore, 60 patients were required in this study. Survival was calculated from the date of initiation using the Kaplan–Meier method.

### RESULTS

During the period August 1995–March 1997 a total of 63 patients were enrolled. One patient did not receive the agent because of rapid progression immediately after registration. This patient was judged as ineligible and excluded from the analysis. The other 62 patients were considered to be eligible and their characteristics are listed in Table 1. There were 43 patients with colon and 19 with rectal carcinoma as the primary site. Forty-eight patients had a prior history of surgical resection. Ten patients had an additional history of adjuvant chemotherapy. All 10 adjuvant chemotherapy patients were treated with a regimen including 5-FU or oral

*© 2000 Cancer Research Campaign*

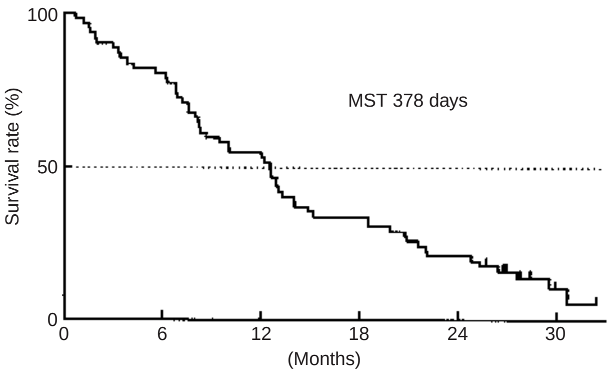

**Figure 1** Overall survival of the 62 eligible patients.

**Table 2** Response results

| | Patients (*n*) | CR | PR | NC | PD | NE | Response rate (%) |
|---|---|---|---|---|---|---|---|
| Overall | 62 | 0 | 22 | 28 | 8 | 4 | 35.5* |
| Colon | 43 | 0 | 15 | 19 | 7 | 2 | 34.9 |
| Rectum | 19 | 0 | 7 | 9 | 1 | 2 | 36.8 |
| Metastatic site | | | | | | | |
| Liver | 40 | 1 | 10 | 20 | 6 | 3 | 27.5 |
| Lung | 28 | 0 | 11 | 15 | 1 | 1 | 39.3 |
| Others | 14 | 1 | 4 | 4 | 2 | 3 | 35.7 |

CR = Complete Response; PR = Partial Response; NC = No Change; PD = Progressive Disease; NE = Not Evaluated. *95% confidence interval, 24.7–47.9%

fluorinated pyrimidines, predominantly UFT. Only one patient had received pelvic radiotherapy.

A total of 271 courses were administered to the 62 patients with a median of four courses. Fifty-three (85%) of the 62 patients were treated as outpatients. The other nine patients received the agent as inpatients because of easier management or patient's preference, which is usual in Japanese clinical trials associated with low hospitalization cost. No patients required dose reduction due to adverse reactions. Compliance was extremely good with an actual administration rate of 97%.

Twenty-two (35%) of the 62 patients achieved PR with a 95% confidence interval of 25–48%. Responses for each of the target sites were 39% in lung, 28% in liver, and 50% in abdominal node metastases (Table 2). Five of the 10 patients with a history of adjuvant chemotherapy achieved PR. There were no significant differences in response rates by actually administered doses per body surface area. Patients administered < 70, < 75, and ≥ 75 mg $m^{-2}$ $day^{-1}$ of the agents, achieved response rates of 44, 30, and 35%, in 16, 23, and 23 patients, respectively. The median time to achieve a 50% reduction of the tumour and median response duration were 37 (23–85) days and 171 (78–389) days, respectively. The median survival time of the 62 patients was 12 months with a 2-year survival rate of 21% (Figure 1).

The most serious adverse reactions during the treatment are listed in Table 3. Major adverse reactions included myelosuppressive and gastrointestinal toxicities, though they were generally mild and no treatment-related deaths occurred. Five (8%) patients developed grade 4 thrombocytopenia, three in the first, of whom one was associated with grade 4 neutropenia, one in the second, and one in the fourth course of the treatment. Grade 4 leukopenia was also seen in two (5%) patients. There was one early death on day 21 caused by hyperosmolar diabetic coma, where the patient had diabetes mellitus before commencement of the treatment. No other grade 4 toxicity occurred during the study. Only one patient developed either grade 3 nausea or grade 3 vomiting and diarrhea. Skin toxicities were rarely seen, with occurence in less than 10% of the patients, except for skin pigmentation which was seen in 18%. Incidence of grade 3 or 4 toxicity tended to be higher in patients administered 70 mg $m^{-2}$ $day^{-1}$ or more than those receiving less than 70 mg $m^{-2}$ $day^{-1}$, 39% vs 13% ($P$ = 0.098) respectively. None of the 53 patients treated as outpatients required hospitalization due to adverse reactions.

**Table 3** Toxicity

| Toxicity | Grade (No. of patients) 1 | 2 | 3 | 4 | Incidence of ≥ Grade 3 (%) |
|---|---|---|---|---|---|
| Haematological | | | | | |
| Leukopenia | 17 | 10 | 1 | 2 | 4.8 |
| Neutropenia | 4 | 11 | 7 | 1 | 12.9 |
| Anaemia | 5 | 11 | 4 | 0 | 6.5 |
| Thrombocytopenia | 5 | 2 | 0 | 5 | 8.1 |
| Non-haematological | | | | | |
| Stomatitis | 8 | 2 | 0 | 0 | – |
| Diarrhoea | 2 | 6 | 1 | 0 | 1.6 |
| Anorexia | 7 | 11 | 3 | 0 | 4.8 |
| Nausea/vomiting | 7 | 4 | 1 | 0 | 1.6 |
| Skin rash | 2 | 4 | 0 | 0 | – |
| Pigmentation | 11 | 0 | 0 | 0 | – |
| Malaise | 9 | 2 | 1 | 0 | 1.6 |

### DISCUSSION

Two major advantages have been reported in oral chemotherapy, one being pharmacoeconomic and the other being patient preference (DeMario et al, 1998). Cost will become a central issue particularly in palliative settings such as chemotherapy for metastatic colorectal carcinoma. In response to issues relating to the administrative cost of this disease, future trends should be directed to outpatient chemotherapy. The issue of patient preference has been reported by Liu et al (1997). The study revealed that more than 90% of the patients with advanced solid malignancies preferred oral agents if they provided comparable efficacy to infusional agents. In the present study, most of the patients were treated as outpatients without requiring hospitalization for adverse reactions. The agent S-1 also exhibited similar efficacy to, for instance, a combination of infusional 5FU plus leucovorin, with less toxicities. These results appeared to fulfill the major preferences for oral agents.

Bioavailability and interpatient biovariability are usually major problems that are required to be elucidated in oral agents. From the in vivo study using rats, the bioavailability of S-1 was found to be 102% with respect to tegafur, though it was 58% and 25% with respect to CDHP and potassium oxonate respectively. In the previous phase I study, sufficient plasma concentration of 5-FU, more than 100 ng $ml^{-1}$, was achieved with the patients treated at the dose and schedule regimen employed in the present study (Taguchi et al, 1997). Interpatient AUC variability appeared to be small with a lower frequency of critical toxicity, which shows a

© 2000 Cancer Research Campaign general correlation with the pharmacodynamics. These two pharmacokinetic parameters provided enough information to elucidate the major problems affecting the efficacy of this oral agent. The present study also revealed clinical activity for colorectal carcinoma with a response rate of 35%, which seemed to be comparable to those in other combination regimens such as 5-FU plus leucovorin (The Advanced Colorectal Cancer Meta-Analysis Project, 1992; Poon et al, 1989; Petrelli et al, 1989; Leichman, 1995). Although myelosuppression of this agent tended to be higher than those of UFT with or without leucovorin, the incidence of grade 3 or 4 toxicities was only less than 13%. The survival rate of the present study, with a median survival time of 12 months, also demonstrated similar results to those in the standard 5-FU and leucovorin regimen. Based on these pharmacokinetic and clinical outcomes, S-1 may provide clinical benefits comparable with intravenous combination regimens.

DPD is known to be the initial and rate-limiting enzyme affecting 5-FU catabolism, converting approximately 90% of administered 5-FU to α-fluoro-β-alanine (Heggie et al, 1987). The importance of this enzyme was first recognized from the critical toxicity in deficient patients (Tuchman et al, 1985; Diasio et al, 1988) followed by circadian rhythm of its activity and chronomodulated therapy (Harris et al, 1990; Levi et al, 1992). Recently, DPD has also been pointed out as a determining factor regarding its sensitivity to 5-FU. Etienne et al 1995) reported that DPD activity in pretreatment tumour tissues correlated to a clinical response with thymidylate synthase activity in patients with head and neck cancer treated with 5-FU-based chemotherapy. This evidence was compounded by a rationale involving a biochemical modulation of 5-FU using a DPD inhibitor, eniluracil (Baccanari et al, 1993; Schilsky et al, 1997). Schilsky et al 1997) reported a 33% response rate using an oral 5-day schedule of 5-FU at 20 mg $m^{-2}$, leucovorin at 50 mg $day^{-1}$, and eniluracil at 20 mg $day^{-1}$. However, the inhibition of DPD by this agent is irreversible, with possible toxicity. As a result, the above regimen was associated with significant neutropenia, which required hospitalization in 10 of the 24 patients registered. In contrast, CDHP contained in S-1 is a reversible DPD inhibitor and our results indicated mostly mild toxicity without hospitalization or cumulative toxicity. Five of the 10 patients with a history of adjuvant chemotherapy containing 5-FU achieved objective responses, suggesting a biochemical modulation effect by CDHP.

Our data, including the previous study, suggest that S-1 achieves similar responses to a standard regimen of 5-FU plus leucovorin and shows the potential of being an alternative to that combination. The agent also exhibited easily manageable toxicity and was readily accepted by our patients. Further investigations of the agent including a randomized trial are warranted.

### ACKNOWLEDGEMENTS

We are grateful to Drs K Ota, S Tsukagoshi, I Nakao, H and Furue, for kind advice, Drs W Koizumi, J Ota, N Hirabayashi and Y Maehara for extramural review and many other participants of the S-1 Cooperative Colorectal Carcinoma Study Group during the study. We also thank T Tahara, M Dantsuji, K Iizuka, K Hoashi,H Anbe and K Okabe for assistance in data collection and analysis. This study was supported by Taiho Pharmaceutical Co Ltd,Tokyo.

### REFERENCES

Advanced Colorectal Cancer Meta-Analysis Project (1992) Modulation of fluorouracil by leucovorin in patients with advanced colorectal cancer. *J Clin Oncol* **10**: 896–903

Baccanari DP, Davis ST, Knick VC and Spector T (1993) 776C85: Effects on the pharmacokinetics and antitumor activity of 5-fluorouracil. *Proc Natl Acad Sci USA* **90**: 11064–11068

DeMario MD and Ratain MJ (1998) Oral chemotherapy: rationale and future directions. *J Clin Oncol* **16**: 2557–2567

Diasio RB, Beavers TL and Carpenter JT (1988) Familial deficiency of dihydropyrimidine dehydrogenase. *J Clin Invest* **81**: 47–51

Etienne MC, Cheradame S, Fischel JL, Formento P, Dassonville O, Renee N, Schneider M, Thyss A, Demard F and Milano G (1995) Response to fluorouracil therapy in cancer patients: The role of tumoral dihydropyrimidine dehydrogenase activity. *J Clin Oncol* **13**: 1663–1670

Harris BE, Song R, Soong SJ and Diasio RB (1990) Relationship between dihydropyrimidine dehydrogenase activity and plasma 5-fluorouracil levels with evidence for circadian variation of enzyme activity and plasma drug levels in cancer patients receiving 5-fluorouracil by protracted continuous infusion. *Cancer Res* **50**: 197–201

Heggie GD, Sommadossi JP, Cross DS, Huster WJ and Diasio RB (1987) Clinical pharmacokinetics of 5-fluorouracil and its metabolites in plasma, urine, and bile. *Cancer Res* **47**: 2203–2206

Houghton JA, Houghton PJ and Wooten RS (1979) Mechanism of induction of gastrointestinal toxicity in the mouse by 5-fluorouracil, 5-fluorouridine, and 5-fluoro-2'-deoxyuridine. *Cancer Res* **39**: 2406–2413

Ikenaka K, Shirasaka T and Kitano S (1979) Effect of uracil on metabolism o 5-fluorouracil in vitro. *Jpn J Cancer Res* **70**: 353–359

Japanese Research Society for Cancer of Colon and Rectum (1994) *General rules for clinical and pathological studies on cancer of the colon, rectum and anus*. 5th edn. Kanehara Syuppan: Tokyo

Japan Society for Cancer Therapy (1993) Criteria for the evaluation of the clinical effects of solid cancer chemotherapy. *J Jpn Soc Cancer Ther* **28**: 101–130

Levi F, Misset JL, Brienza S, Adam R, Metzger G, Itzakhi M, Caussanel JP, Kunstlinger F, Lecouturier S, Descorps-Declere A, Jasmin C, Bismuth H and Reinberg A (1992) A chronopharmacologic phase II clinical trial with 5-fluorouracil, folinic acid, and oxaliplatin using an ambulatory multichannel programmable pump. *Cancer* **69**: 893–900

Leichman CG, Fleming TR, Muggia FM, Tangen CM, Ardalan B, Doroshow JH, Meyers FJ, Holcombe RF, Weiss GR, Mangalik A and Macdonald JS (1995) Phase II study of fluorouracil and its modulation in advanced colorectal cancer: A Southwest Oncology Group Study. *J Clin Oncol* **13**: 1303–1311

Liu G, Franssen E, Fitch MI and Warner E (1997) Patient preference for oral versus intravenous palliative chemotherapy. *J Clin Oncol* **15**: 110–115

Malik ST, Talbot D, Clarke PI, Osborne R, Reznek R, Wrigley PF and Slevin ML (1990) Phase II trial of UFT in advanced colorectal and gastric cancer. *Br J Cancer* **62**: 1023–1025

Pazdur R, Lassere Y, Rhodes V, Ajani JA, Sugarman SM, Patt YZ, Jones DV Jr, Markowitz AB, Abbruzzese JL, Bready B and Levin B (1994) Phase II trial of uracil and tegafur plus oral leucovorin: An effective oral regimen in the treatment of metastatic colorectal carcinoma. *J Clin Oncol* **12**: 2296–2300

Petrelli N, Douglass HO Jr, Herrera L, Russell D, Stablein DM, Bruckner HW, Mayer RJ, Schinella R, Green MD, Muggia FM, Megibow A, Greenwald ES, Bukowski RM, Harris J, Levin B, Gaynor E, Loutfi A, Kalser MH, Barkin JS, Benedetto P, Woolley PV, Nauta R, Weaver DW and Leichman LP for the gastrointestinal tumor study group (1989) The modulation of fuorouracil with leucovorin in metastatic colorectal carcinoma: A prospective randomized phase III trial. *J Clin Oncol* **7**: 1419–1426

Poon MA, O'Connel MJ, Moertel CG, Wieand HS, Cullina SA, Everson LK, Krook JE, Mailliard JA, Laurie JA, Tschetter LK and Wiesenfeld M (1989) Biochemical modulation of fluorouracil: Evidence of significant improvement of survival and quality of life in patients with advanced colorectal carcinoma. *J Clin Oncol* **7**: 1407–1418

Sakata Y, Ohtsu A, Horikoshi N, Sugimachi K, Mitachi Y and Taguchi T (1998) Late phase II study of novel oral fluoropyrimidine anticancer drugs S-1 (1M tegafur–0.4M gimestat–1M otastat potassium) in advanced gastric cancer patients. *Eur J Cancer* **34**: 1715–1720

Schilsky R, Bukowski R, Burris H, Crawford J, Hochster H, O'Rourke M, Steinfeldt H, Doucette M, Levin J and Hohneker J (1997) A phase II study of a five day regimen of 5-fluorouracil (5-FU) plus GW776 (776C85) with or without

© 2000 Cancer Research Campaign leucovorin in patients with metastatic colorectal cancer. *Proc Am Soc Clin Oncl* **16**: 271a (abst 961)

Shirasaka T, Nakano K, Takechi T, Satake H, Uchida J, Fujioka A, Saito H, Okabe, H Oyama K, Takeda S, Unemi N and Fukushima M (1996) Antitumor activity of 1M tegafur-0.4M 5-chloro-2,4-dihydroxypyridine-1M potassium oxonate (S-1) against human colon carcinoma orthotopically implanted into nude rats. *Cancer Res* **56**: 2602–2606

Shirasaka T, Shimamoto Y and Fukushima M (1993) Inhibition by oxonic acid of gastrointestinal toxicity of 5-fluorouracil without loss of its antitumor activity in rats. *Cancer Res* **53**: 4004–4009

Sugimachi K, Maehara Y, Horikoshi N, Shimada Y, Sakata Y, Mitachi Y, Taguchi T and The S-1 Gastrointestinal Cancer Study Group (1999) An early phase II study of oral S-1, a newly developed 5-fluorouracil derivative for advanced and recurrent gastrointestinal cancers. *Oncology* **57**: 202–210

Takiuchi H and Ajani JA (1998) Uracil-tegafur in gastric carcinoma: a comprehensive review. *J Clin Oncol* **16**: 2877–2885

Tatsumi K, Fukushima M, Shirasaka T and Fujii S (1987) Inhibitory effects of pyrimidine, barbituric acid and pyridine derivatives on 5-fluorouracil degradation in rat liver extracts. *Jpn J Cancer Res* **78**: 748–755

Takechi T, Nakano K, Uchida J, Mita A, Toko K, Takeda S, Unemi N and Shirasaka T (1997) Antitumor activity and low intestinal toxicity of S-1, a new formulation of oral tegafur, in experimental tumor models in rats. *Cancer Chemother Pharmacol* **39**: 205–211

Taguchi T, Inuyama Y, Kanamaru R, Hasegawa K, Akazawa S, Niitani H, Furue H, Kurihara M, Ota K, Suga S, Ariyoshi Y, Takai S, Shimoyama T, Toge T, Takashima S, Sugimachi K, Hara Y, Fujita H, Kimura K, Saito T, Tsukagoshi S and Nakao I (1997) Phase I study of S-1. *Jpn J Cancer Chemother* **24(15)**: 2253–2264

Tuchman M, Stoeckeler JS, Kiang DT, O'Dea RF, Ramnaraine ML and Mirkin BL (1985) Familial pyrimidinemia and pyrimidinuria associated with severe fluorouracil toxicity. *N Engl J Med* **313**: 245–249

*© 2000 Cancer Research Campaign* 

 *© 2000 Cancer Research Campaign*
